# The relationship between preoperative anxiety and acute pain and nausea-vomiting in children after surgery

**DOI:** 10.3389/fped.2026.1956966

**Published:** 2026-09-11

**Authors:** Aytül Çoban, Zerrin Çiğdem, Melike Yavaş Çelik

**Affiliations:** 1 Hospital of Harran University, Şanlıurfa, Türkiye; 2 Department of Nursing, Istanbul Topkapı University, Faculty of Health Sciences, İstanbul, Türkiye; 3 Department of Midwifery, Gaziantep University, Faculty of Health Sciences, Gaziantep, Türkiye

**Keywords:** acute pain, anxiety, child - age, nausea, vomiting

## Abstract

**Objective:**

The aim of this study is to evaluate the relationship between preoperative anxiety and acute pain and nausea-vomiting in children.

**Method:**

In this study, FLACC Pain Scale, modified Yale Preoperative Anxiety Scale and BARF Nausea Scale Score were used. The statistical analysis of our study was performed using SPSS version 25.0 program. Shapiro–Wilk test was used to evaluate the normal distribution of variables. Descriptive analysis used mean and standard deviation values for presentation purposes.

**Results:**

A total of 77 patients were included in the study. The mean age of the patients was 9.84 ± 2.52 years and 74.03% were male. 24.68% of the children were attending second grade. 37.66% of the children had undergone surgery before and 46.75% had been hospitalized before. The mean hospitalization time was 3.37 ± 3.58. It was found that there was a relationship between the FLACC Pain Scale, Yale Preoperative Anxiety Scale and Barf Nausea Scales, and as a result of the regression analysis, anxiety had a mediating role in the occurrence of pain and nausea.

**Conclusion:**

These results showed that preoperative anxiety is associated in the prevention and treatment of postoperative pain and nausea-vomiting in children.

## Introduction

Considering the surgical processes, children face many health problems. Anxiety is one of these problems. Acute pain, nausea and vomiting that occur after surgery due to anxiety are serious problems. Children are generally more vulnerable than adults, and their individual control is weaker than adults. Anxiety occurs more frequently in children, especially in the younger age group (less than 15 years old) (1).

Anxiety is generally a form of symbolic sign. Anxiety can trigger many problems. For this reason, in order to increase the comfort level of children after surgery and to help them cope with this situation, detailed evaluation of pain and nausea and vomiting should be made frequently and the treatment and care process should be planned according to the results of the evaluation (2).

Acute pain occurs suddenly and can be defined as a sign of disease. The severity of the pain and how long it will last cannot be known with certainty. Acute pain, sometimes reaching unbearable levels, can cause many negative consequences by affecting other parts, systems and functions in the body. For this reason, it is known that acute pain is not a type of pain that should be ignored and that it is very difficult to cope with this situation (3). Acute pain, in addition to physical complaints in the child, also reveals some behavioral/mental states such as restlessness (4). Anxiety is considered one of the most important causes of acute pain after surgery. It is suggested that high levels of anxiety and stress in the child further increase acute pain. This situation causes acute pain and other negative and undesirable conditions to develop (5).

Nausea and vomiting are side effects of pain and anesthesia and are a condition that negatively affects children's quality of life (6). Postoperative nausea and vomiting may reveal negative psychosocial problems such as patient dissatisfaction and discomfort. It may also cause physical consequences such as nausea and vomiting, fluid-electrolyte imbalance, aspiration pneumonia, esophageal rupture, and wound dehiscence. These situations may delay the child's discharge from the hospital and therefore increase healthcare costs (7).

Surgery is a challenging process for children, and this process must be managed effectively by parents, healthcare professionals, and other team members working in this field. Anxiety that can be effectively managed before surgery can prevent acute pain and nausea and vomiting that children may experience after surgery (6). Therefore, the aim of this study is to evaluate the relationship between preoperative anxiety and acute pain and nausea-vomiting in children.

### Question of research

1. What is the role of anxiety in children before surgery on acute pain and nausea and vomiting after surgery?

## Method

Type of Research: Relationship searching and descriptive.

### Population and sample of the research

The G*Power 3.1.9.4 program was used to determine the sample size. The sample was calculated by taking into account the article written by Wang & Kain (2000) (8), which has similar characteristics to the study. The effect size was taken as (*ρ* = 0.32) and the sample size was found to be 71 with 80% power. A total of 431 children were admitted to the Pediatric Surgery Clinic for surgery between 12.09.2022 and 20.01.2023, when the study was conducted. For the sample, 77 children who met the inclusion criteria listed below were included in the sample.

#### Criteria for inclusion in the study

- Be over 7 years old (required to be able to communicate while collecting data, This age group was chosen to ensure compatibility with the scales used for data collection)
- Being hospitalized for surgery (clinic admission could be due to some different diseases)
- Volunteering to participate in the study
- Ability to read and write Turkish (some immigrants and Kurdish citizens in the region where the hospital where the research is conducted cannot speak Turkish)
- No diagnosed neurological or psychiatric disease

#### Exclusion criteria from the study

- Becoming a refugee
- Staying in hospital for less than 48 h after surgery (Nausea and vomiting should have been evaluated within the 48th hour)
- Being brought in for emergency surgery
- Experiencing the recent death of a loved one
- Having previous surgical experience

### Data collection

The study was conducted between October 2022 and January 2023 in children aged 7 and older who underwent elective surgery in the 18-bed Pediatric Surgery Clinic. Collecting the data took 15-20 min.

### Tools

Data were collected by the Descriptive Characteristics Form, Yale Preoperative Anxiety Scale, FLACC Pain Scale, and Baxter Animated Retching Faces/Baxter(BARF) Nausea Scale.

#### Descriptive characteristics form

This form was created by scanning studies related to the study (9–11). It was created by descriptive characteristics of the children [Body Mass Index (BMI), height, body weight, gender, age, education level, economic status of the family, number of siblings]

#### Yale preoperative anxiety scale

The scale is suitable for children aged 8-12. The Turkish validity and reliability study of the scale was conducted by Hatipoğlu et al. In 2019, Cronbach's alpha value was found to be 0.85. The scale consists of 22 items and 5 sub-dimensions. The subscales are Activity (4 items), Vocal Reactions (6 items), Emotional Expression (4 items), Degree of Arousal (4 items), and Parent-Related Reactions (4 items) (12). While scoring the scale, the score obtained from each sub-dimension is divided by the highest score that can be obtained from that sub-dimension and the results are summed. This result is divided by the total number of sub-dimensions and multiplied by 100. As a result of this process, a total score ranging from 23.33 to 100 is obtained. A higher score from the scale indicates higher anxiety (12). The Modified Yale Preoperative Anxiety Scale was filled out by the investigator 1 h before the surgery, while the patient was at the patient's bed with his/her parent next to him/her. children were evaluated twice. Analyzes were made by taking the average of two evaluations.

#### FLACC pain scale

FLACC pain assessment scale is a scale used to evaluate postoperative pain in pediatric patients. It was developed by Merkel and colleagues in 1997 (13). Turkish language validity was done by Şenayli et al. (2006). Its Turkish translation was made in 2006. The original scale was developed for children aged 2-7, while the Turkish validity and reliability study was conducted on children aged 1-9. Scales with a wider age range may be used in future studies.

The two-way cross-stratification method was used to examine the agreement among pediatric surgeon, anesthetist and nurse raters independently of each other, and it was reported that the Kappa Value was found to be significant (13). The scale consists of a total of 5 sections evaluated between 0 and 2. These sections; Face, Legs, Activity, Cry, Consolability. Application of the scale requires observing the child every 10 min for 50 min and giving a score between 0 and 2 for each section. The total score varies between 0 and 10. A score of 0 indicates that the child is calm and comfortable, a score of 1-3 indicates that the child is slightly uncomfortable, scores between 4 and 6 indicate that the child is in moderate pain, and scores between 7 and 10 indicate that the child is significantly uncomfortable and in pain. When the total pain score is 4 or above, additional analgesics are recommended (13).

In this study, all children were evaluated for pain by observing the Flacc pain scale immediately after being taken to their postoperative rooms, and by observing them 5 times at 10-minute intervals for a total of 50 min. Analyzes were carried out by taking the average of these evaluations. The children's parents were also with the children during this evaluation process. In the practice of the clinic where our study was conducted, routine analgesia was not applied to every child, and analgesia prescribed by the physician was administered to children who complained of pain after the first ten minutes and whose scale score was four or more.

#### BARF nausea scale

It was developed by William Baxter in 2010 to evaluate postoperative nausea in children over the age of seven. The scale is a visual evaluation scale consisting of six facial expressions scored between 0 and 10. Turkish validity and reliability was conducted by Şişman et al. in 2016. There are a total of six items in the BARF Scale and six facial expressions that define each item (14).

This scale was applied at the 2nd hour after surgery. Children were asked to choose which face shape suits them at that moment using the BARF Scale. Since no patient was administered antiemetics within the last half hour before the evaluation, nausea and vomiting were not evaluated again.

### Statistical analysis of data

Statistical analyzes were performed with SPSS version 25.0 program. The suitability of the variables for normal distribution was examined with the Shapiro–Wilk test. Mean and standard deviation values were used when presenting descriptive analyses. Frequency and percentage values of the variables were used when presenting categorical variables. Relationships between quantitative variables were examined with Pearson Correlation Analysis. Correlation coefficient r*, very strong correlation between 0.81 and 1.0; Strong correlation between 0.60 and 0.79; moderate correlation between 0.40 and 0.59; Between 0.20 and 0.39 was considered a weak correlation, and between 0 and 0.19 was considered a very weak correlation. Cases where the *p*-value was below 0.05 were considered statistically significant.

### Ethical aspects

The study was granted ethical clearance by the institution's General Human Research Ethics Committee. The research was conducted with children who were allowed to participate in the research by legally protected individuals (mother, father, grandfather, uncle). The research was conducted by the Principles of the Declaration of Helsinki. To protect the interests of the participants, the researcher complied with ethical standards throughout the study.

## Results

The average age of the children was 9.84 ± 2.52 years. It was found that 74.03% (*n* = 57) of the children, were boys and 24.68% (*n* = 19) of the children were attending second grade. The average body weight of the children was 30.78 ± 9.88 kg and the average height of the children was 132.19 ± 10.92 cm. It was determined that 44.16% (*n* = 34) of the mothers were illiterate and 87.01% (*n* = 67) were not working. It was determined that 32.47% (*n* = 25) of the fathers had secondary education level and 64.94% (*n* = 50) were working. It was observed that the income of 71.43% (*n* = 55) of the children's families did not cover their expenses and the average number of siblings was 5.17 ± 2.20 (Table 1).

**Table 1:** Distribution of descriptive characteristics of children.

Data		Mean ± S.D *n* = 77	Median Min-Max (%)
Age		9,84 ± 2,52	9 (7–16)
Sex	FemaleS	20	25,97
	Male	57	74,03
School	No school	6	7,79
	1.class	12	15,58
	2.class	19	24,68
	3.class	9	11,69
	4.class	12	15,58
	5.class	5	6,49
	6.class	7	9,09
	7.class	5	6,49
	8.class	1	1,30
	9.class	1	1,30
Weight (kg)		30,78 ± 9,88	28 (18–65)
Height (cm)		132,19 ± 10,92	130 (110–160)
Body mass index		17,21 ± 2,85	16,85 (12,5–25,4)
Mother's education level	Illiterate	34	44,16
	Literate	22	28,57
	Secondary education	7	9,09
	High school education University education	13	16,88
		1	1,30
Mother's working status	No	67	87,01
	Yes	10	12,99
Father's education level	Illiterate	7	9,09
	Literate	20	25,97
	Secondary education High school education University education	25	32,47
		22	28,57
		3	3,90
Father's working status	No	27	35,06
	Yes	50	64,94
Income	Income is lower than expenses	55	71,43
	Income equals expenses	20	25,97
	Income exceeds expenses	2	2,60
Number of siblings (Mean)		5,17 ± 2,20	5 (2–12)

The total Yale Preoperative Anxiety Scale and the total score averages of the subscales were as follows; Activity: 1.97 ± 1.16, Vocal reactions: 2.53 ± 1.54, Emotional Expression: 2.36 ± 1.12, Degree of Arousal: 2.31 ± 1.17, Parent-Related Reactions: 2.23 ± 1.05 and the total points: 11.42 ± 5.42 (Table 2).

**Table 2:** Children's mean scores of the Yale preoperative anxiety scale, FLACC pain scale, and BARF nausea scale.

Scale		Mean ± S.D *n* = 77	Median (Min. Max) %=100
Yale Preoperative Anxiety Scale	Activity	1,97 ± 1,16	2 (1–4)
	Vocal Reactions	2,53 ± 1,54	2 (1–6)
	Emotional Expression	2,36 ± 1,12	3 (1–4)
	Degree of Arousal	2,31 ± 1,17	3 (1–4)
	Parent-Related Reactions	2,23 ± 1,05	2 (1–4)
	Total score	11,42 ± 5,42	11 (5–22)
FLACC Pain Scale	Calm and confortable	5	6,49
	Slightly uncomfortable	9	11,69
	Moderate pain	36	46,75
	Significantly Uncomfortable and in pain	27	35,06
	Total score	5,68 ± 2,88	5 (0–10)
BARF			
Nausea Scale	Total score	2,23 ± 3,21	2 (0–10)

According to the FLACC Pain Scale classification, 6.49% (*n* = 5) of the children were calm and comfortable, 11.69% (*n* = 9) were slightly uncomfortable, 46.75% (*n* = 36) were moderately uncomfortable. 35.06% (*n* = 27) were significantly uncomfortable and the mean scale score was 5.68 ± 2.88 (Table 2).

The mean BARF Nausea Scale score of the children at the 2nd hour after surgery was evaluated as 2.23 ± 3.21 (Table 2).

A positive and moderate (r = 0.602, *p* < 0.001) statistically significant relationship was detected between the Yale Preoperative Anxiety Scale and FLACC Pain Scale scores. A positive and moderate (r = 0.414, *p* < 0.001) statistically significant relationship was found between Yale Preoperative Anxiety Scale and BARF Nausea Scale. A positive and moderate (r = 0.384, *p* < 0.001) statistically significant relationship was found between FLACC Pain Scale and BARF Nausea Scale (Table 3).

**Table 3:** Relationship between scales.

		YALE	FLACC	BARF
FLACC Pain Scale	*r	**0,602**	1	
	p	**<0,001**	-	
BARF Nausea Scale	r	**0,414**	**0,384**	1
	p	**<0,001**	**<0,001**	-

* r: pearson correlation coefficient.

*p* < 0.05.

There is a moderately positive and significant correlation between the total and subscale scores of the Modified Yale Preoperative Anxiety Scale and the FLACC Pain Scale score (*p* < 0.05). There is also a weakly significant positive correlation between the total and subscale scores of the Modified Yale Preoperative Anxiety Scale and the BARF Nausea Scale score (*p* < 0.05). However, the relationship between the BARF Nausea Scale score and parental-related reactions is not significant (Table 4).

**Table 4:** Relationship between modified yale preoperative anxiety scale and its subdimensions, and FLACC pain and BARF nausea scales.

		Total YALE	Activity	Vocalization	Emotional Expression	Degree of Arousal	Parent-Related Reactions
FLACC	r p	0,571 **<0,001**	0,519 **<0,001**	0,546 **<0,001**	0,518 **<0,001**	0,510 **<0,001**	0,448 **<0,001**
BARF	r p	0,266* 0,019	0,242* 0,034	0,251* 0,028	0,253* 0,026	0,246* 0,031	0,194 0,090

* r: pearson correlation coefficient.

*p* < 0.05.

## Discussion

In this study, which we conducted to evaluate the relationship between preoperative anxiety in children and postoperative acute pain and nausea-vomiting, a strong relationship was found. A similar study revealed that there is a relationship between preoperative anxiety and postoperative pain, and that different factors, along with anxiety, play a role in the formation of postoperative pain (15). In another study, it was determined that there was a statistically significant positive relationship between pre- and postoperative anxiety score averages and pain score averages (16). According to the FLACC Pain Scale classification, 6.49% (*n* = 5) of the children were calm and comfortable, 11.69% (*n* = 9) were slightly uncomfortable, 46.75% (*n* = 36) were moderately uncomfortable. 35.06% (*n* = 27) were significantly uncomfortable and the mean scale score was 5.68 ± 2.88 (Şenaylı et al., 2006). A positive and moderate (r = 0.602, *p* < 0.001) statistically significant relationship was detected between the Modified Yale Preoperative Anxiety Scale score and the FLACC Pain Scale of Postoperative Pain in Children score.

Anxiety causes differences in physiological and psychological variables in children. Physiological changes cause changes in cardiac and respiratory functions due to the suppression of immunity and increased sensitivity to pain due to the release of stress hormones such as cortisol (17). As a result of children experiencing anxiety and fear in the preoperative period, the following psychological problems occur: nausea, restlessness, and angry and aggressive behavior behavioral and physiological traumas (18, 19).

Studies have revealed that especially children experience nausea and vomiting in the second hour after surgery, that the rate of postoperative nausea and vomiting in children may be 5% in infancy and 20% in the preschool period, and that it may reach even higher rates as age progresses, especially in adolescence (18, 20, 21). The average age of the children in this study was 9.84 ± 2.52 and it was determined that scored of BARF Nausea Scale of the children was 2.23 ± 3.21. Based on the self-reported average scores of these children, who are old enough to express themselves, the BARF Nausea scale indicated that they experienced strong feelings of nausea.

According to the evaluation of the scale, this score was interpreted as children not having many problems with nausea and vomiting (14). Additionally, a positive and moderate (r = 0.414, *p* < 0.001) statistically significant relationship was detected between the Yale Preoperative Anxiety Scale and the BARF Nausea Scale (BARF).

Wiggins and Foster (2007) evaluated the pain of children after tonsillectomy and adenoidectomy operations. As a result of this study, it was found that children's pain rates were mostly between 3.1-3.3 and that 64.7% of children woke up at night due to their pain and 52.9% of children experienced nausea (22).

## Conclusion

Preoperative anxiety generally reflected children's fear, anxiety and stress levels regarding surgery. These emotions can affect children's perception of pain, their ability to tolerate pain, and increase their physiological and behavioral reactions to pain. This study found a strong correlation between preoperative anxiety and acute postoperative pain, nausea, and vomiting. This finding proved how valuable it is to reduce anxiety and stress before surgery. For this reason, it is important to prepare children before surgery and adapts to the hospital environment. Family-centered care, therapeutic play, holistic care and primary nursing care may be beneficial in preventing anxiety in children and helping the child adapt to the situation it is experiencing.

## Data Availability

The raw data supporting the conclusions of this article will be made available by the authors, without undue reservation.
